# Inside-Out Regulation of ICAM-1 Dynamics in TNF-α-Activated Endothelium

**DOI:** 10.1371/journal.pone.0011336

**Published:** 2010-06-28

**Authors:** Jaap D. van Buul, Jos van Rijssel, Floris P. J. van Alphen, Mark Hoogenboezem, Simon Tol, Kees A. Hoeben, Jan van Marle, Erik P. J. Mul, Peter L. Hordijk

**Affiliations:** 1 Sanquin Research and Landsteiner Laboratory, Department of Molecular Cell Biology, Academic Medical Center, University of Amsterdam, Amsterdam, The Netherlands; 2 Department of Cell Biology and Histology, Academic Medical Center, Amsterdam, The Netherlands; Max Planck Institute of Molecular Cell Biology and Genetics, Germany

## Abstract

**Background:**

During transendothelial migration, leukocytes use adhesion molecules, such as ICAM-1, to adhere to the endothelium. ICAM-1 is a dynamic molecule that is localized in the apical membrane of the endothelium and clusters upon binding to leukocytes. However, not much is known about the regulation of ICAM-1 clustering and whether membrane dynamics are linked to the ability of ICAM-1 to cluster and bind leukocyte integrins. Therefore, we studied the dynamics of endothelial ICAM-1 under non-clustered and clustered conditions.

**Principal Findings:**

Detailed scanning electron and fluorescent microscopy showed that the apical surface of endothelial cells constitutively forms small filopodia-like protrusions that are positive for ICAM-1 and freely move within the lateral plane of the membrane. Clustering of ICAM-1, using anti-ICAM-1 antibody-coated beads, efficiently and rapidly recruits ICAM-1. Using fluorescence recovery after photo-bleaching (FRAP), we found that clustering increased the immobile fraction of ICAM-1, compared to non-clustered ICAM-1. This shift required the intracellular portion of ICAM-1. Moreover, biochemical assays showed that ICAM-1 clustering recruited beta-actin and filamin. Cytochalasin B, which interferes with actin polymerization, delayed the clustering of ICAM-1. In addition, we could show that cytochalasin B decreased the immobile fraction of clustered ICAM-1-GFP, but had no effect on non-clustered ICAM-1. Also, the motor protein myosin-II is recruited to ICAM-1 adhesion sites and its inhibition increased the immobile fraction of both non-clustered and clustered ICAM-1. Finally, blocking Rac1 activation, the formation of lipid rafts, myosin-II activity or actin polymerization, but not Src, reduced the adhesive function of ICAM-1, tested under physiological flow conditions.

**Conclusions:**

Together, these findings indicate that ICAM-1 clustering is regulated in an inside-out fashion through the actin cytoskeleton. Overall, these data indicate that signaling events within the endothelium are required for efficient ICAM-1-mediated leukocyte adhesion.

## Introduction

Leukocytes use integrins to firmly adhere to activated endothelium through adhesion molecules such as VCAM-1 and ICAM-1 prior to extravasation. Over the past years, the endothelium is increasingly recognized to actively contribute to the adhesion and transmigration of leukocytes [1]. Binding of leukocyte integrin αL/Mβ2 induces ICAM-1 clustering, resulting in downstream signalling in the endothelium [2]–[5]. This intracellular signalling induced into the endothelium regulates rearrangement of the actin cytoskeleton. We and others have shown increased stress fiber formation after ICAM-1 antibody crosslinking [6], [7]. These changes in the actin cytoskeleton imply an important role for Rho-like small GTPases [8]. In agreement with this notion, various studies have shown that RhoA, which induces stress fibers and contractility, is activated downstream from ICAM-1 clustering [6], [9], [10]. Blocking RhoA activity by C3 toxin or overexpression of a dominant negative mutant (RhoA-T19N) prevents antibody-mediated and monocyte-induced clustering of ICAM-1, monocyte adhesion and subsequent stress fiber formation [11]. These data indicate that active RhoA is involved in ICAM-1 function. In addition, upon clustering, either by antibody-mediated crosslinking or by adhesion of THP-1 cells, ICAM-1 is incorporated into detergent-insoluble membrane domains, i.e. lipid rafts [12]. Interestingly, this shift to the insoluble fraction is not inhibited by cytochalasin B, an inhibitor of actin polymerization, suggesting that this response is independent from RhoA.

Pioneering work from Barreiro and co-workers showed that endothelial cells actively protrude sheets of membrane around an adherent leukocyte, referred to as docking structures [13]. These authors showed that several actin-binding proteins are present in these structures, such as vinculin, paxillin, alpha-actinin, VASP and ERM-proteins. From other reports, it became clear that the intracellular domain of ICAM-1 plays an essential role in its function. Deleting the intracellular domain of ICAM-1 decreases transendothelial migration (TEM) of leukocytes [14], [15]. This was further underscored by a study by Sans et al., in which they expressed ICAM-1 in CHO cells that lack endogenous ICAM-1 [16], which was sufficient to reconstitute TEM. However, expression of a C-terminally deleted mutant lf ICAM-1 severely impaired efficient TEM. Recently, using expression in Cos cells, Oh and colleagues defined the RKIKK motif within the intracellular domain of ICAM-1 as critical for ICAM-1 function [17]. These data point to the importance of ICAM-1 in TEM. ICAM-1 exists as a dimer and can freely diffuse in the plasma membrane [18]. Upon clustering by antibody-mediated crosslinking, ICAM-1 reduces its lateral mobility, most likely because of increased association of the intracellular tail to actin cytoskeleton-binding proteins [19]. Although all these efforts, it is still not known which signals and molecules regulate ICAM-1 function and mobility.

In this study, we analysed ICAM-1 dynamics by using fluorescence recovery after photobleaching (FRAP) prior to and following ICAM-1 clustering. In addition, the adhesive function of ICAM-1 was studied under flow. We show that ICAM-1 function is regulated by the connection of its C-terminal intracellular domain to downstream, cytoskeleton-regulating proteins myosin-II and the small GTPase Rac1, but not Src-like kinases. Our data provide evidence for control of ICAM-1 function in leukocyte adhesion in an inside-out fashion through the modulation of ICAM-1 lateral mobility and linkage to the actin cytoskeleton.

## Materials and Methods

### Reagents and Abs

Monoclonal antibody (mAb) against ICAM-1 was purchased from R&D Systems (Minneapolis, MN); Polyclonal Abs against ICAM-1 (for WB, no.7891) was purchased from Santa Cruz Biotechnology (Santa Cruz, CA). ICAM-1-ALEXA 647 mAb (Clone 15.2) was purchased from Serotec (Oxford, UK). The GFP mAb was purchased from Invitrogen (Carlsbad, CA). Actin antibody and Cytochalasin B were purchased form Sigma-Aldrich (Zwijndrecht, the Netherlands). Recombinant Tumor-Necrosis-Factor (TNF)-α was purchased from PeproTech (Rocky Hill, NJ); Texas-Red Phalloidin, ALEXA 488-labelled GαM-Ig, ALEXA 568-labelled GαM-Ig, ALEXA 568-labelled GαR-Ig, ALEXA 488-labelled GαR-Ig and ALEXA 488-labelled streptavidin secondary Abs were from Molecular Probes (Leiden, The Netherlands) and all used in a 1:500 dilution. Jasplakinolide was purchased from Merck KGaA (Darmstadt, Germany).

### Expression Vectors

ICAM-1-wt-GFP was a kind gift from Dr. Sanchez-Madrid (Madrid, Spain). The C-terminal deletion mutant was generated by deleting the 27 aa at the C-terminus.

### Cell cultures, treatments and transfections

Primary HUVECs (Human Umbilican Vein Endothelial cells) were obtained from Cambrex (East Rutherford, NJ) and cultured as described previously [10]. Endothelial cells were activated with 10 ng/ml TNF-α overnight to mimic inflammation. All cell lines were cultured or incubated at 37°C at 5% CO_2_. The HL60 pro-myelocytic cell line was routinely cultured in IMDM, supplemented with 10% fetal bovine serum (Sigma). Where indicated, DMSO-differentiated HL60 cells were used. Differentiation to a neutrophil-like phenotype was achieved by including 1.3% DMSO in the medium for 3–5 days which resulted in increased expression of the LFA-1 and Mac-1 integrins [20]. Cos7 and Hela cells were maintained in IMDM with 10% fetal bovine serum. Cells were transiently transfected with the expression vectors indicated in each experiment according to the manufacturer's protocol using LipofectAMINE PLUS (Invitrogen) or Fugene6 (Roche, Basel, Swiss).

### Immunofluorescence (IF)

Cells were cultured on FN-coated-glass cover slips, fixed and immunostained with indicated primary Abs as described previously [7]. Subsequent visualization was performed with Alexa-conjugated secondary Abs (Invitrogen). F-actin was visualized with fluorescently-labeled Phalloidin (Invitrogen). Images were recorded with a ZEISS LSM510 confocal microscope with appropriate filter settings. Crosstalk between the different channels was avoided by use of sequential scanning.

### Scanning electron microscopy

Transfected cells were grown on glass coverslips, fixed in 2.5% glutaraldehyde/PBS for 30 min at room temperature, and processed for scanning EM as described previously [10]. Cells were examined on a scanning electron microscope (model 820; JEOL) at 15 kV.

### Antibody-coated beads

3- or 10-µm polystyrene beads (Polysciences, Inc.) were pretreated with 8% glutaraldehyde overnight, washed five times with PBS, and were incubated with 300 µg/ml ICAM-1 mAb (R&D systems) or polyclonal rabbit Ab (Santa Cruz) according to the manufacturer's protocol. Magnetic goat-anti-mouse IgG Ab-coated Dynabeads (Invitrogen) were coated with ICAM-1 or IgG isotype control Abs according to the manufacturers' protocol.

### Adhesion under flow

HUVECs were cultured on FN-coated glass cover slips, incubated with inhibitors as indicated, together with 1 µg/mL αICAM-1 antibody-coated beads for 30 minutes. Next, shear flow is introduced at 0.25dyn/cm^2^ (200 µL/minute) for 5 minutes, and then increased to 1.25dyn/cm^2^ for 5 minutes. Next, flow was increased every 5 minutes with 1.25dyn/cm^2^ up to 6.25dyn/cm^2^ (5 mL/minute) finally. Images were collected and the number of adherent leukocytes was counted per field of view. Note that the inhibitors were included in the medium and thus were present throughout the experiment.

### Immunoprecipitation and Western blot analysis

Cells were grown to confluency on FN-coated dishes (50 cm^2^), washed twice gently with ice-cold Ca^2+^-and Mg^2+^-containing PBS and lysed in 300 µL of lysis buffer (25 mM Tris, 150 mM NaCl, 10 mM MgCl2, 2 mM EDTA, 0.02% SDS, 0.2% deoxycholate, 1% NP-40, 0.5 mM orthovanadate with the addition of fresh protease-inhibitor-cocktail tablets (Boehringer) pH 7.4). After 10 minutes on ice, cell lysates were collected and pre-cleared for 30 minutes at 4°C with Streptavidin beads (Sigma-Aldrich, 15 µl for each sample). The supernatant, cleared by centrifugation (14.000*g, 5 minutes at 4°C) was incubated with 15 µl protein Streptavidin beads that were coated with the biotin-tagged S27D or the control peptide, for 1 h at 4°C under continuous mixing. The beads were washed 3 times in lysis buffer and proteins were eluted by boiling in SDS-sample buffer containing 4% 2-mercaptoethanol (Bio-Rad). The samples were analyzed by 10% SDS-PAGE. Proteins were transferred to 0.45 µm nitro-cellulose (Schleicher and Schnell Inc., NH) and the blots were first blocked with 5% (w/v) low-fat milk in TBST for 1 h, subsequently incubated at room temperature with the appropriate Abs for 1 h, followed by 30 minutes incubation with HRP-conjugated secondary Abs at RT. Between the various incubation steps, the blots were washed 5 times with TBST and finally developed with an enhanced chemiluminescence (ECL) detection system (Amersham).

### Statistics

Student-T test was used to calculate statistical significance.

## Results

**Figure 1:**
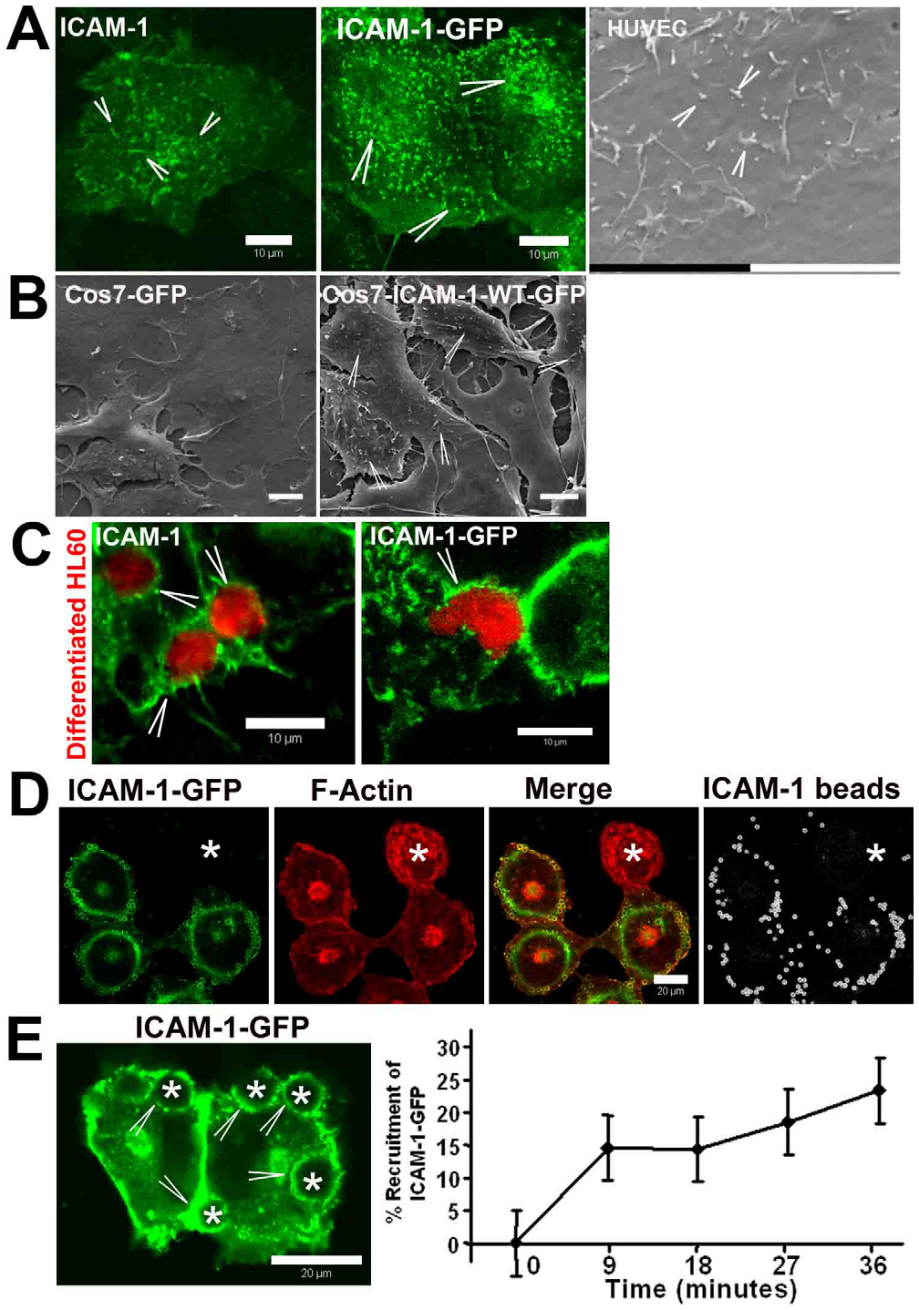
ICAM-1 clusters to leukocyte adhesion sites. (**A**) Left panel shows localization of endogenous ICAM-1 in green on endothelial filopodia (Arrowheads). Middle panel shows localization of ICAM-1-GFP, expressed in HeLa cells on filopodia. Right panel shows scanning electron microscopy (SEM) recording of TNF-α-pretreated HUVECs visualising small filopodia protruding out of the apical plane of the membrane (arrowheads). Bars, 10 µm (**B**) Cos7 cells expressing GFP (left panel) or ICAM-1-GFP (right panel) were cultured on glass cover slips and analyzed by SEM. ICAM-1-GFP but not GFP only-expressing Cos7 cells showed multiple filopodia (arrowheads). Bar, 10 µm. (**C**) TNF-α-pretreated HUVECs were cultured on glass cover slips and calcein-red labelled differentiated HL60 cells were allowed to adhere for 30 minutes. Arrowheads show recruitment of ICAM-1 to adherent HL60 cells. Images show ICAM-1 in green and HL60 cells in red. Right panel shows ICAM-1-GFP-transfected HeLa cells, cultured on glass cover slips and calcein-red labelled differentiated HL60 cells adhered for 30 minutes. Bar, 10 µm. (**D**) ICAM-1-GFP-transfected Cos7 cells were cultured on glass covers and αICAM-1-antibody coated beads (size 3 µm) adhered for 30 minutes. Arrowheads show recruitment of ICAM-1-GFP to adhered beads. Images show ICAM-1-GFP in green, F-actin in red, merge in yellow and the beads in white. Note that the beads specifically adhered to ICAM-1-GFP expressing cells. The asterisk shows non-transfected cell with no bead adhesion. Bar, 20 µm. (**E**) ICAM-1-GFP (green) is expressed in HeLa cells and recruited (arrowheads) to αICAM-1-antibody coated beads (size 10 µm; asterisks). Quantification of ICAM-1 recruitment on the right shows that ICAM-1-GFP is recruited to 25% of the beads after 36 minutes. Data are mean ± SEM from four experiments.

In the course of our studies on ICAM-1 dynamics in endothelial cells, we noticed that ICAM-1 on the surface of endothelial cells as well as ICAM-1-GFP, transfected in HeLa cells, localizes to small, fibrillar structures (Fig. 1A). Subsequent scanning electron microscopy analysis showed that the surface of the apical membrane of the endothelium forms small filopodia-like protrusions (Fig. 1A), reminiscent of the ICAM-1-positive fibrils, identified by fluorescent microscopy. Interestingly, transfection of ICAM-1-GFP into Cos7 cells, which normally do not expose filopodia [21], resulted in the induction of multiple filopodia (Fig. 1B). These data showed that ICAM-1 induces the formation of dorsal filopodia. Live-cell imaging of transfected HeLa cells showed that ICAM-1-GFP-positive dorsal filopodia are highly motile (Movie S1). ICAM-1 is well recognized for its adhesive function in binding leukocytes, and we and others have shown ICAM-1 to be concentrated in ring-like structures surrounding adherent leukocytes. In contrast, there is relatively little knowledge about the dynamics of ICAM-1 recruitment upon binding to an adhered leukocyte. To study this, leukocytes were allowed to adhere to TNF-α-treated endothelial cells and ICAM-1 distribution was analyzed. Using HL60 cells, we found ICAM-1 recruitment to sites of leukocyte adhesion (Fig. 1C). The same effect was observed when leukocytes adhered to HeLa cells expressing ICAM-1-GFP (Fig. 1C). To mimic leukocyte-induced ICAM-1 clustering, we used polystyrene beads that were coated with anti-ICAM-1 antibodies [10]. These antibodies efficiently block leukocyte adhesion and thus target an essential epitope in the extracellular domain of the ICAM-1 molecule [7]. The anti-ICAM-1-antibody coated beads only adhered to ICAM-1-GFP expressing cells and recruited ICAM-1-GFP to sites of bead adhesion in a similar fashion as the leukocytes (Fig. 1D and 1E). Additional experiments showed that ICAM-1-GFP co-localized with endogenous ICAM-1 in endothelial cells and clustered around adhered anti-ICAM-1-antibody coated beads (Fig. S1A and S1B). To study the kinetics of ICAM-1 recruitment, the recruitment of ICAM-1-GFP to sites of bead adhesion was recorded in real-time using confocal laser scanning microscopy. ICAM-1-GFP was transfected into HeLa-cells to avoid dimerization with endogenous ICAM-1 in endothelial cells [18]. These experiments showed that after 30 minutes, approximately 25% of the anti-ICAM-1-antibody coated beads that adhered to ICAM-1-GFP-positive cells recruited ICAM-1-GFP (Fig. 1E).

**Figure 2:**
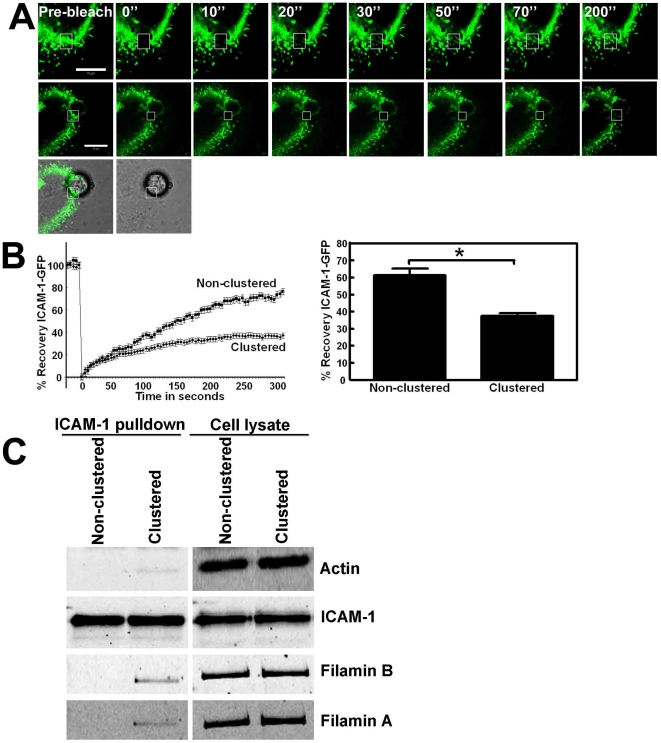
Lateral mobility of ICAM-1 depends on its C-terminal domain. (**A**) ICAM-1-GFP is expressed in HeLa cells and FRAP was measured. Box in the upper panel depicts the FRAP region (square) and the recovery in time after every 10 seconds. Image at the right shows recovery after 200 seconds. Middle panel shows ICAM-1-GFP FRAP that is clustered around an adherent bead, coated with ICAM-1 antibodies. Lower image shows the merge of the green channel and DIC, in which the bead is visible. Bars, 10 µm. (**B**) Quantification of the FRAP data show decreased ICAM-1-GFP mobility following clustering. Graph on the left shows ICAM-1-GFP FRAP in time and graph on the right shows the difference in ICAM-1 mobility at t = 300 seconds. ICAM-1 was clustered using αICAM-1 antibody-coated beads. ICAM-1 FRAP was significantly reduced upon clustering. Experiment is carried out 6 times. Data are mean ± SEM. *p<0.01. (**C**) TNF-α-pretreated HUVECs were cultured on FN-coated surfaces and anti-ICAM-1-coated magnetic beads were allowed to adhere to the endothelium for 30 minutes, resulting in clustering of ICAM-1, after which the cells were lysed. As a control, the beads were added to endothelial cell lysates. Lysate conditions prevent ICAM-1 from clustering and these samples are therefore marked as Non-clustered. Next, using a magnetic pen, the beads were isolated from the cell lysates, washed and analyzed using Western blotting. The images on the left show that the magnetic beads efficiently precipitated ICAM-1 and that ICAM-1 clustering resulted in the recruitment of actin and filamin A and B. Images on the right show the expression of indicated proteins in the cell lysates.

To focus in more detail on the dynamics of ICAM-1 distribution in the plasma membrane, we used a FRAP (Fluorescence Recovery After Photobleaching)-based approach. ICAM-1-GFP, expressed in HeLa cells, was bleached within a small area of the cell and the recovery of ICAM-1-GFP was monitored in real time. The data showed that levels of ICAM-1-GFP were restored to approximately 60% within 5 minutes after bleaching (Fig. 2A and 2B). This data shows that 30–35% of ICAM-1-GFP resides in the so-called ‘immobile fraction’. Similar results were obtained when ICAM-1-GFP was transiently transfected into endothelial cells (data not shown). Interestingly, recovery of clustered ICAM-1-GFP, induced by the anti-ICAM-1-antibody coated beads, was significantly reduced (37±4%), compared to non-clustered ICAM-1-GFP (61±2%; Fig. 2B). To calculate the immobile fraction of ICAM-1-GFP, fluorescence recovery was measured when a plateau phase at 5 minutes was reached. These data showed that the immobile fraction of ICAM-1-GFP increased over 2-fold upon clustering (23±4% for not clustered ICAM-1-GFP compared to 62±5% for clustered ICAM-1-GFP). This indicates that clustering drives ICAM-1 into an immobile fraction.

We previously showed that the actin-binding protein filamin binds to clustered rather than to non-clustered ICAM-1. To study if, in parallel, actin is recruited to clustered ICAM-1, we used magnetic beads, coated with the ICAM-1 antibody, to pull-out associating proteins. To do this, the beads were allowed to adhere and to cluster ICAM-1 on intact, TNF-α-treated HUVEC for 30 minutes. Next, the beads were isolated by extraction from cell lysates as described in Materials and Methods. As a control, the magnetic beads were added post-lysis, preventing ICAM-1 clustering. Western blot analysis showed that the clustering of ICAM-1 induced binding to actin (Fig. 2C). As a positive control, we showed that filamin A and B bind selectively to clustered ICAM-1, in line with our previous findings [22]. Filamin and actin were also recruited to exogenous ICAM-1-GFP (Fig. S1C). Moreover, by limiting the ICAM-1 clustering time, we could show that actin and filamin were recruited in a time-dependent manner to ICAM-1. After five minutes of clustering, no actin and filamin was associated to ICAM-1. However, after 20 minutes of clustering, actin and filamin were both precipitated with ICAM-1 (Fig. S1D). These data showed that clustering of ICAM-1 promotes binding to the actin cytoskeleton, possibly through filamin A or B.

**Figure 3:**
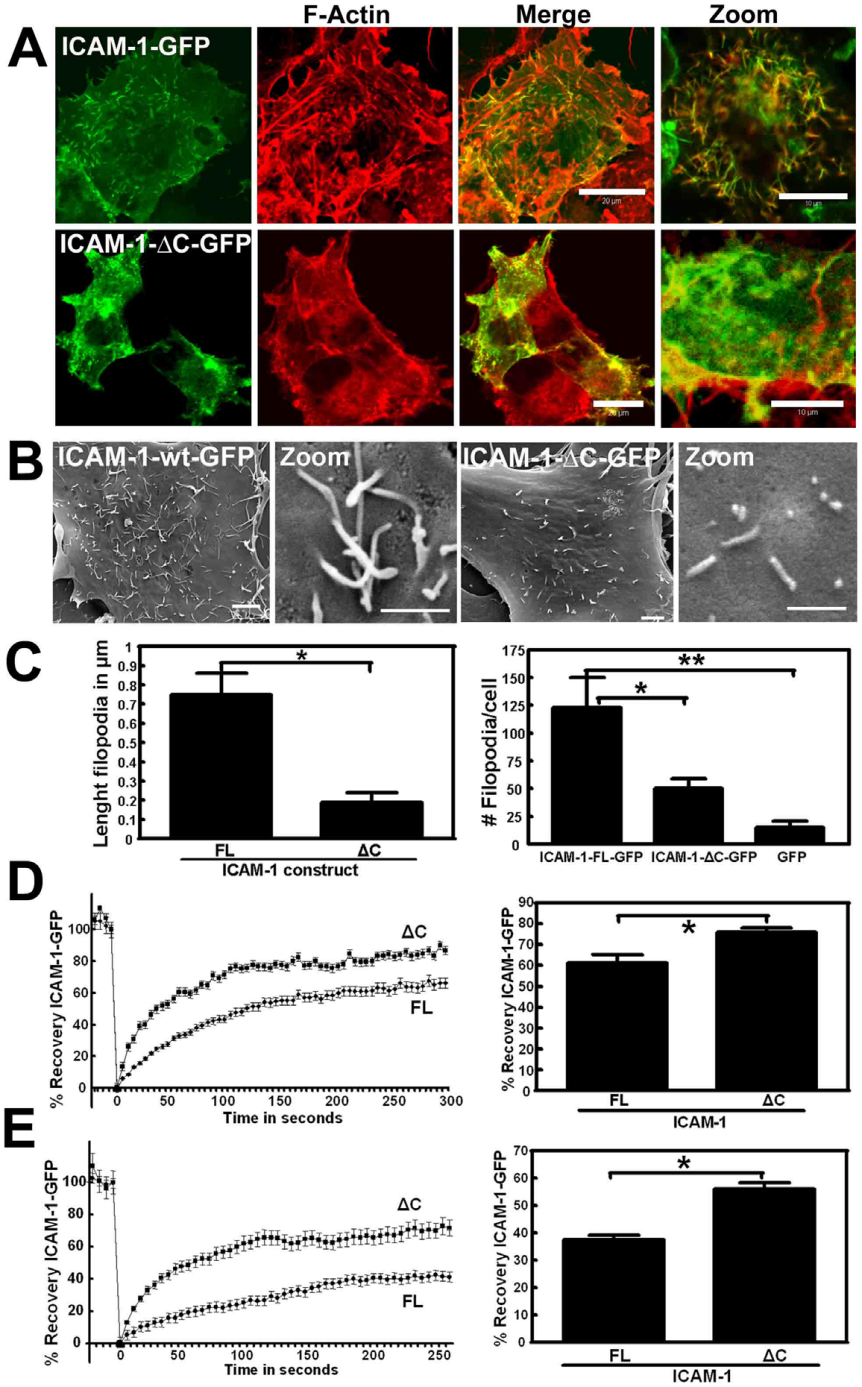
Requirement of the intracellular domain of ICAM-1 for clustering. (**A**) ICAM-1-GFP full length or a construct lacking the intracellular tail (ICAM-1-ΔC) were transfected in HeLa cells and cultured on glass covers. Full-length ICAM-1 localizes to filopodia whereas the ΔC-mutant shows a more dispersed distribution. Images show ICAM-1 staining in green, F-actin in red and merge in yellow. Image on the right is a magnification of the merge. Bars, 20 µm. (**B**) ICAM-1-GFP full length or ΔC was transfected in Cos7 cells that lack basal filopodia and images were taken using a scanning electron microscope. Zoom shows filopodia in full-length or ICAM-1 ΔC expressing Cos7 cells, Bar, 1 µm. (**C**) Quantification of filopodia in Cos7 cells shows that ICAM-1 full length induces filopodia with an average length of 750nm. The ΔC-mutant shows smaller and fewer filopodia. Bar graph shows number of filopodia per Cos7 cell. Experiment is done three times. Data are mean ± SEM. *p<0.01. **p<0.001. (**D**) Full length and ΔC ICAM-1-GFP are expressed in HeLa cells. Left panel shows FRAP in time and right panel shows FRAP quantification after 5 minutes of bleaching. ICAM-1-ΔC recovers significantly faster than full length ICAM-1. Experiment is done four times. Data are mean ± SEM. *p<0.05. (**E**) Full length and ΔC ICAM-1-GFP are expressed in HeLa cells and ICAM-1 clustering was induced through αICAM-1 antibody-coated beads. Left panel shows FRAP in time and right panel shows FRAP quantification after 5 minutes of bleaching. Clustered ICAM-1-ΔC recovers significantly faster than full length. Experiment is done four times. Data are mean ± SEM. *p<0.05.

To determine whether the intracellular tail of ICAM-1 is required for the shift of clustered ICAM-1 to the immobile fraction, we used an ICAM-1-GFP mutant that lacks the intracellular domain (ICAM-1-ΔC-GFP). ICAM-1-ΔC-GFP localized only to a limited extent to filopodia-like structures, as compared to full-length ICAM-1 (Fig. 3A). Using scanning electron microscopy, we found that Cos7 cells transfected with full length ICAM-1 expressed large numbers of filopodia with an approximate length of 750 nm (Fig. 3B and 3C). The number of dorsal filopodia in the Cos7 cells that were transfected with ICAM-1-ΔC was significantly reduced (Fig. 3B and 3C). In addition to that, the average length of these filopodia was reduced to approximately 200 nm (Fig. 3C). Interestingly, FRAP-analysis showed that the mobility of non-clustered ICAM-1-ΔC was significantly increased compared to full length ICAM-1 (Fig. 3D). Moreover, a similar difference was observed when ICAM-1 was clustered with αICAM-1-antibody coated beads, i.e. increased recovery after bleaching of ICAM-1-ΔC compared to ICAM-1 full length (Fig. 3E). In line with this, we found that 37±7% of ICAM-1-ΔC resides in the immobile fraction as compared to 65±6% for full length ICAM-1. These results underscore the importance of the intracellular tail in the shift of ICAM-1 to the immobile fraction.

**Figure 4:**
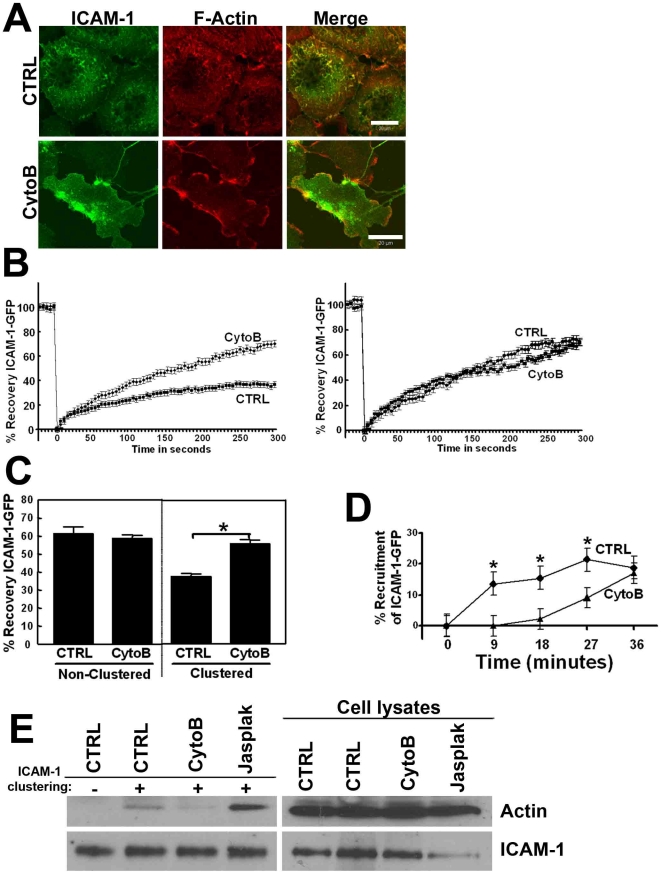
Actin polymerization is required for the shift of ICAM-1 to the immobile fraction. (**A**) HUVECs were treated with Cytochalasin B (CytoB; 5 µg/mL) for 10 minutes to block actin polymerization, fixed and analyzed by fluorescent microscopy. CytoB prevents ICAM-1 localization to filopodia. Images show ICAM-1 staining in green and F-actin in red. Merge shows co-localization of ICAM-1 and F-actin. Bar, 20 µm. (**B**) ICAM-1-GFP is expressed in HeLa cells and treated or not with CytoB. ICAM-1 is clustered as described in B. Left panel shows no difference in FRAP of non-clustered ICAM-1 upon CytoB treatment. Panel at the right shows increased ICAM-1 FRAP when actin polymerization was blocked. Experiment is done three times. Data are mean ± SEM. *p<0.05. (**C**) Quantification of clustered and non-clustered ICAM-1 shows that ICAM-1-GFP recruitment upon clustering is delayed upon CytoB treatment but not in non-clustered conditions. Experiment is repeated 3 times and data are mean ± SEM. *p<0.05. (**D**) ICAM-1-GFP is expressed in HeLa cells and recruited to αICAM-1-antibody coated beads as described under 1E. Quantification graph shows that ICAM-1-GFP recruitment is delayed when actin polymerization is blocked. Experiment is repeated 3 times and data are mean ± SEM. *p<0.05. (**E**) TNF-α-pretreated HUVECs were cultured on FN-coated surfaces and prior to anti-ICAM-1-coated magnetic beads-induced clustering, cells were pretreated with CytoB or jasplakinolide (10 µM). Next, beads were allowed to adhere to the endothelium for 30 minutes, and samples were processed as described in the legend of figure 2C. Panels on the left show magnetic beads pull down. ICAM-1 was precipitated equally under all conditions. Clustering of ICAM-1 induced the link to actin, which was prevented by CytoB pre-treatment. Stabilizing actin filaments by jasplakinolide promoted the link of ICAM-1 with actin upon clustering. Panels on the right show the expression of indicated proteins in the cell lysates.

Clustering of ICAM-1 resulted in increased accumulation of F-actin at sites of binding of anti-ICAM-1-antibody coated beads or adhesion of leukocytes [23]. Carman and co-workers already showed by using cytochalasin B (CytoB) that F-actin is required for the induction of apical cups, surrounding adherent lymphocytes [24]. Our data showed that interfering with actin polymerization using CytoB drastically reduced the number of ICAM-1-positive dorsal filopodia (Fig. 4A). Next, we studied the role of actin polymerization in the dynamics of ICAM-1-GFP. FRAP analysis showed that basal dynamics of ICAM-1-GFP were not altered following CytoB treatment (Fig. 4B). Interestingly, FRAP of ICAM-1-GFP at sites of adhesion was significantly increased upon inhibition of actin polymerization (Fig. 4B). Recalculation of the immobile fraction showed that CytoB significantly reduced the immobile fraction from 63±7% to 34±6%. Figure 4C showed the quantification of the FRAP. In addition, when αICAM-1-antibody coated beads were used to cluster ICAM-1-GFP, the recruitment of ICAM-1 to sites of adhesion was delayed in CytoB-treated cells as compared to untreated cells (Fig. 4D). Nevertheless, after approximately 36 minutes of beads adhesion, the number of beads that recruited ICAM-1 was similar in CytoB-treated and in control cells. To study if blocking of actin polymerization affected the linkage of ICAM-1 with actin, induced by clustering, a pull-out assay using anti-ICAM-1-Ab coated magnetic beads was performed, which showed that CytoB treatment prevented the interaction of ICAM-1 with actin upon ICAM-1 clustering (Fig. 4E). Interestingly, stabilizing actin filaments by jasplakinolide promoted the interaction of ICAM-1 with actin upon clustering (Fig. 4E). Thus, actin polymerization is required for ICAM-1 mobility and for efficient recruitment of ICAM-1 to sites of adhesion.

**Figure 5:**
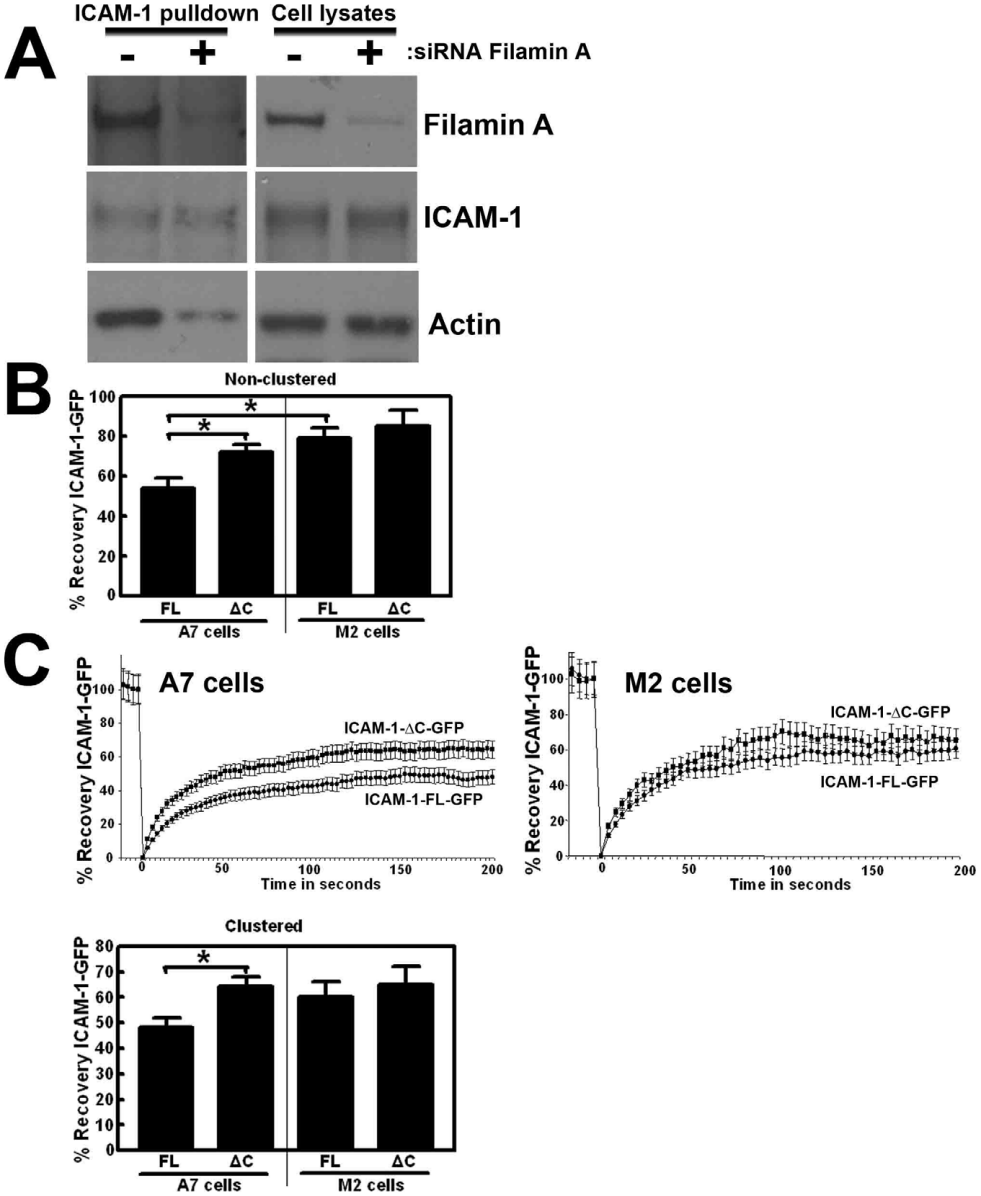
ICAM-1 requires filamin and its intracellular tail for the actin link and its motility. (**A**) TNF-α-pretreated HUVECs were cultured on FN-coated surfaces and filamin expression was reduced using siRNA, according to Kanters et al. [22]. Next, anti-ICAM-1-coated magnetic beads were allowed to adhere to the endothelium for 30 minutes, and samples were processed as described in the legend of figure 2C. Panels on the left show magnetic beads pull down. ICAM-1 was precipitated equally in both conditions. Clustering of ICAM-1 induced the link to actin, which was prevented by silencing Filamin A. Panels on the right show the expression of indicated proteins in the cell lysates. (**B**) Full length (FL) and ΔC ICAM-1-GFP are expressed in M2 and A7 cells. Panel shows FRAP quantification. The data show that the recovery of full length ICAM-1 is faster in the absence of filamin, e.g. in M2 cells. ICAM-1-ΔC-GFP FRAP is independent on the presence of filamin. Experiment is done three times. Data are mean ± SEM. *p<0.05. (**C**) Cells were transfected as described in B. ICAM-1-Ab coated beads were allowed to adhere for 30 minutes to cluster ICAM-1. FRAP was measured at sites of bead adhesion in A7 (left panel) and M2 cells (right panel). Lower panel shows FRAP quantification. The data show that ICAM-1 requires filamin and its intracellular tail to shift to the immobile fraction upon clustering. Experiment is done four times. Data are mean ± SEM. *p<0.05.

We have previously shown that filamin links ICAM-1 to the actin cytoskeleton [22]. In addition, reduction of filamin by siRNA prevented the interaction of ICAM-1 with actin upon clustering (Fig. 5A). These data indicate that filamin plays an important role in the clustering-induced ICAM-1-actin association. To study if filamin is also required in ICAM-1 motility, ICAM-1-GFP is expressed in M2 melanoma cells. These cells lack the expression of filamin and have been widely used in studies on actin regulation [25], [26]. As a control, filamin has been reconstituted in A7 melanoma cells. FRAP analysis of non-clustered ICAM-1-GFP showed increased FRAP in M2 cells, indicating that filamin is required for ICAM-1 motility (Fig. 5B). To study the importance of the intracellular tail of ICAM-1 for its motility, we expressed ICAM-1-ΔC-GFP into M2 and A7 cells and analyzed ICAM-1 motility by FRAP. Our data showed that no difference in the motility of ICAM-1-ΔC-GFP was measured in the presence or absence of filamin (Fig. 5B). To study the role of filamin after ICAM-1 clustering, beads coated with ICAM-1 Ab were used. FRAP analysis showed reduced motility of full length ICAM-1-GFP in A7 cells, compared to the motility of ICAM-1-ΔC-GFP (Fig. 5C). Interestingly, no difference in motility was measured when ICAM-1-full length or ΔC were expressed in M2 cells (Fig. 5C). In addition, these experiments have been repeated in endothelial cells, in which filamin A expression was reduced using siRNA. Similar results were obtained in these cells (data not shown). Together, these data indicate that ICAM-1 needs its intracellular tail and filamin for the mobility upon clustering.

**Figure 6:**
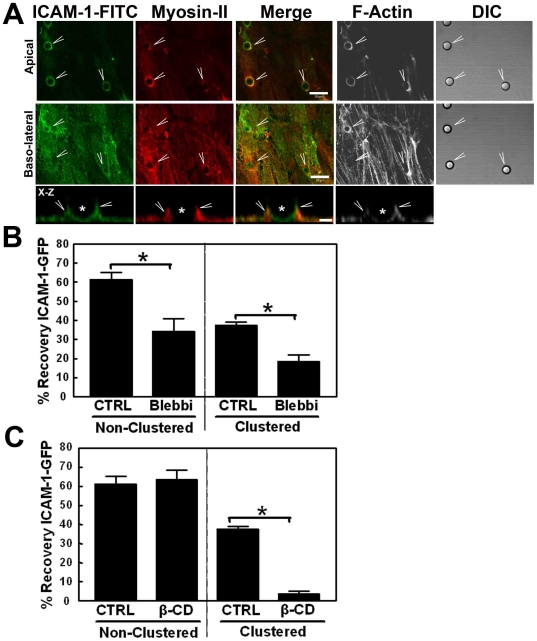
Myosin and lipid raft formation are required for the shift of ICAM-1 to the immobile fraction. (**A**) TNF-α-pretreated HUVECs were cultured on glass covers and images show ICAM-1 staining in green using ICAM-1-FITC to avoid cross-talk with the beads, myosin-II in red, merge of ICAM-1 and myosin-II in yellow, F-actin in white and DIC is included to depict the beads (arrowheads). Myosin-II together with ICAM-1 is clustered around the αICAM-1-antibody coated beads that were allowed to adhere for 30 minutes. Bar, 20 µm. Lower panel shows X-Z projection of ICAM-1 in green and myosin-II in red (arrowheads) around the adhered αICAM-1-antibody coated beads (asterisk). White depicts F-actin. Bar, 5 µm. (**B**) Cells were pretreated with 8 µM Blebbistatin to inhibit myosin-II for 30 minutes (Blebbi). ICAM-1-GFP FRAP was analyzed as described above and showed that Blebbistatin significantly decreased the FRAP of non-clustered and clustered ICAM-1-GFP. Experiment is done three times. Data are mean ± SEM. *p<0.05. (**C**) Cells were pretreated with 5mM methyl-β-cyclodextrin for 30 minutes (β-CD). ICAM-1-GFP FRAP was analyzed as described above and showed that β-CD did not affect ICAM-1-GFP FRAP of non-clustered ICAM1-GFP but significantly reduced the FRAP of clustered ICAM-1-GFP. Experiment is done three times. Data are mean ± SEM. *p<0.01.

Myosins are actin-based motors that regulate actin dynamics [27]. Myosin-II co-localized with ICAM-1 at sites of αICAM-1 antibody-coated beads (Fig. 6A). Inhibiting myosin-II using blebbistatin reduced ICAM-1-GFP dynamics, irrespective of whether ICAM-1 was clustered or not (Fig. 6B). Tilghman and Hoover showed that ICAM-1 shifts to lipid raft-rich membrane domains upon clustering [12]. Preventing the formation of lipid rafts by chelating cholesterol with methyl-β-cyclodextrin (β-CD) reduced the dynamics of clustered ICAM-1-GFP significantly (Fig. 6C). Interestingly, FRAP of non-clustered ICAM-1 was unaltered (Fig. 6C). These data, together with results from the ICAM-1-GFP FRAP analysis on CytoB-treated cells indicate that the clustering of ICAM-1 depends on membrane composition and is controlled by proteins that are involved in cytoskeletal remodeling.

**Figure 7:**
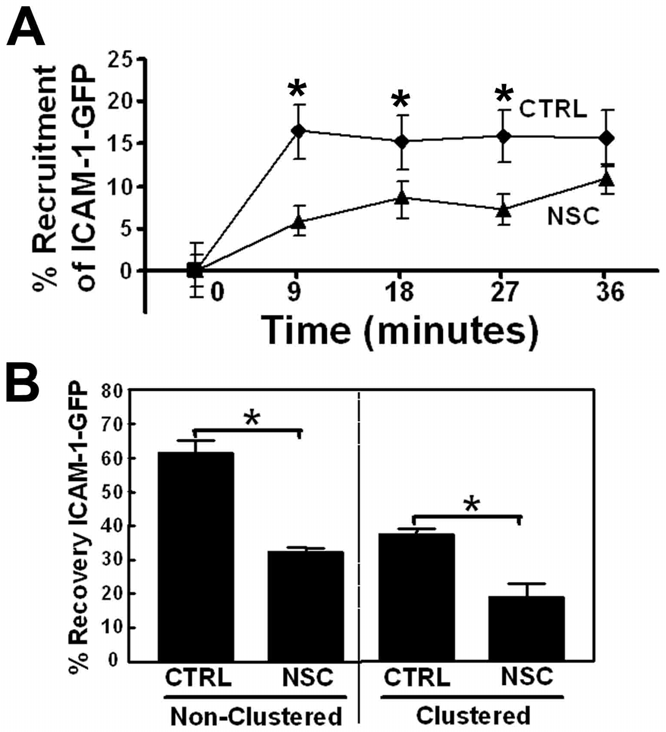
Rac1 activity is required for optimal ICAM-1 dynamics. (**A**) Cells were pretreated with 100 µM NSC-23766 (NSC) for 30 minutes. Quantification of ICAM-1 recruitment using αICAM-1 antibody coated beads shows that ICAM-1-GFP recruitment is reduced upon inhibition of Rac1 activity by NSC. Experiment is repeated 3 times and data are mean ± SEM. *p<0.05. (**B**) ICAM-1-GFP FRAP was analyzed as described in Materials and Methods and showed that NSC reduced FRAP of non-clustered and clustered ICAM-1-GFP. Experiment is done three times. Data are mean ± SEM. *p<0.05.

The small GTPase Rac1 regulates the remodelling of the actin cytoskeleton and induces membrane ruffles [28]. We recently showed that Rac1 is activated upon clustering of ICAM-1 ([10]; Fig. S2A). To elucidate the role of Rac1 activity in ICAM-1 dynamics, we tested the Rac1-GEF inhibitor NSC-23766 on the kinetics of ICAM-1 clustering. Using Trio-GEF-D1, a catalytic active mutant of the exchange factor Trio, we showed that the inhibitor indeed blocked Rac1 activity (Fig. S2B), as was also shown by others [29]. Rac1 inhibition reduced the clustering of ICAM-1-GFP to αICAM-1-antibody coated beads by approximately 50% (Fig. 7A). Next, we assessed the effects of this inhibitor on ICAM-1-GFP dynamics by FRAP analysis. Interestingly, NSC-23766 significantly reduced the dynamics of non-clustered ICAM-1-GFP (Fig. 7B). This indicates that basal Rac1 activity is involved in ICAM-1 mobility in the membrane. In addition, the recovery of clustered ICAM-1-GFP was also significantly reduced upon Rac1 inhibition (Fig. 7B). These data underscore the importance of Rac1 signaling controlling the dynamics of non-clustered as well as clustered ICAM-1.

**Figure 8:**
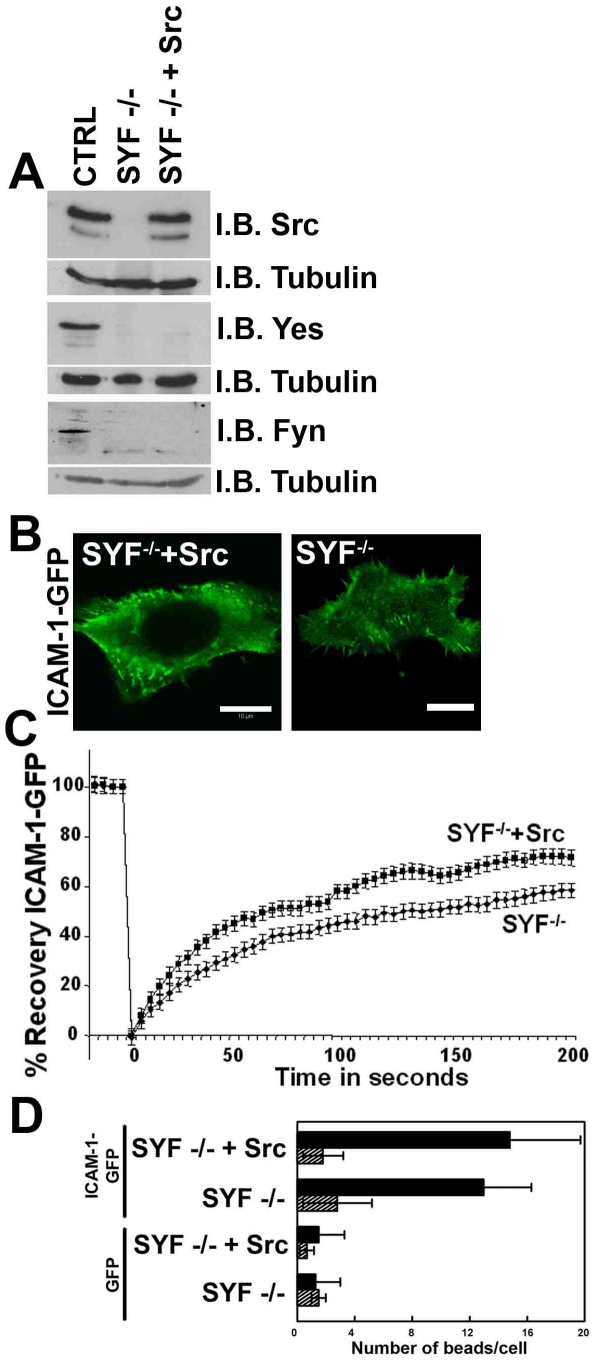
Src-kinase has no effect on ICAM-1 dynamics. (**A**) Analysis of Src, Yes and Fyn-kinase (SYF)-deficient (SYF^-/-^) or SYF^-/-^ with reconstituted Src (SYF^-/-^+Src) or control (CTRL) mouse embryonic fibroblast (MEF) cells by western blotting using antibodies against Src, Yes or Fyn. Tubulin was used for protein loading control. (**B**) Immunofluorescent images show localization of ICAM-1-GFP in filopodia in SYF^-/-^ or SYF^-/-^+Src MEF cells. (**C**) Graph shows that the expression of Src had only minor effect on the ICAM-1 FRAP in the SYF^-/-^ cells. (**D**) αICAM-1 antibody coated beads (size 3 µm) were allowed to adhere for 30 minutes on ICAM-1-GFP transfected SYF^-/-^ or SYF^-/-^+Src cells. Number of beads per ICAM-1-GFP-positive cell was counted. No difference in adhesion of these beads the MEF cells is measured. Experiment was carried out three times.

A key molecule that transmits ICAM-1-induced intracellular signals is Src-kinase [30], [31]. To assess the role of Src in ICAM-1 dynamics, we used Src-, Yes- and Fyn-kinase-deficient (SYF^-/-^) mouse embryonic fibroblast (MEF) cells or SYF^-/-^ MEFs stably re-expressing Src (SYF^-/-^+Src) (Fig. 8A). ICAM-1-GFP was transfected into these cells and fluorescent analysis showed that ICAM-1-GFP was localized in filopodia-like structures in both cell types, albeit more clearly in the Src-reconstituted MEF cells (Fig. 8B). FRAP-analysis showed that the dynamics of non-clustered ICAM-1-GFP depended only minimally on the presence of Src (Fig. 8C). Moreover, depletion of Src did not affect the adhesion of anti-ICAM-1-antibody coated beads to ICAM-1-GFP-positive MEFs (Fig. 8D). In addition, the use of the classical inhibitors of Src-kinase PP1 and PP2 did not affect the adhesion of anti-ICAM-1-antibody coated beads to endothelial cells (data not shown). These data indicate that Src-kinase plays only a minor role in the regulation of the dynamics of ICAM-1.

**Figure 9:**
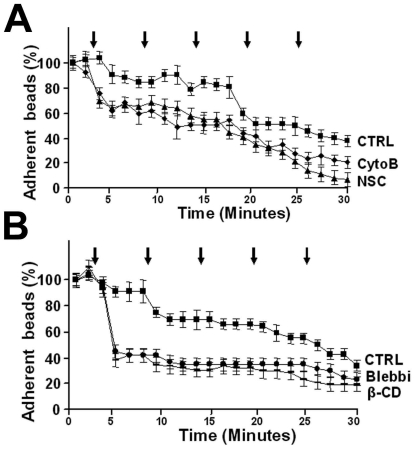
Quantification of ICAM-1 adhesion. (**A**) αICAM-1-antibody coated-beads were allowed to pre-adhere to TNF-α-stimulated endothelium for 5 minutes. Experiment was carried out as described in Materials and Methods. Briefly, low shear was introduced (0.25dyn/cm^2^) and flow was increased stepwise with 1.25dyn/cm^2^ at the time points, indicated with arrows. The final shear corresponded to 6.25dyn/cm^2^. The αICAM-1-antibody coated beads detached from the endothelium when shear increased. Pre-treatment of the endothelium with cytochalasin B (CytoB) or NSC-23766 (NSC) showed a steeper decline of detached beads upon increased shear flow. Experiment is done three times. Data are mean ± SEM. *p<0.05. (**B**) Experiment carried out as described under A. Pre-treatment of the endothelial cells with either blebbistatin (Blebbi) or cyclodextrin (β-CD) show a decrease in adhesion of the beads to the endothelium, already after the first increase in shear flow. Experiment is done three times in duplicate. Data are mean ± SEM. *p<0.01.

**Figure 10:**
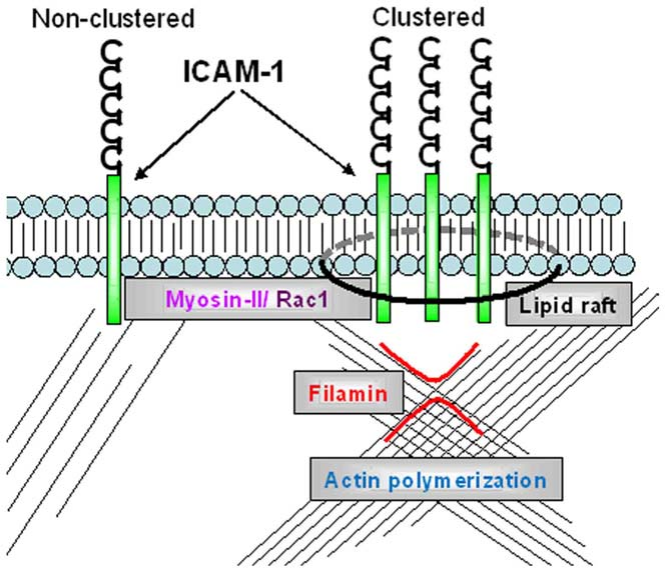
Schematic overview of ICAM-1 dynamics. ICAM-1 dynamics require Rac1 and myosin-II activity. Upon clustering, actin polymerization and lipid raft formation play a pivotal role. These signalling modules all affect the adhesive capacity of ICAM-1 under flow conditions.

Finally, we tested if the lateral mobility of ICAM-1 affects its adhesive function under flow. The inhibitors used needed to be present throughout the experiment. Therefore, anti-ICAM-1 antibody-coated beads were used instead of leukocytes, since the inhibitors may affect the function of the leukocytes. The beads were allowed to adhere to the endothelium for 20 minutes, followed by incubation with the inhibitors for 10 minutes. Then flow was introduced, which gradually increased in time, as described in Materials and Methods. These studies showed that increasing the flow-rate resulted in a decreased adhesion of αICAM-1 antibody-coated beads to the endothelium, indicating that flow negatively regulates ICAM-1 function (Fig. 9A). Subsequent blocking of actin polymerization or Rac1 activation indicated that these are both needed for ICAM-1 function under flow (Fig. 9A). Using blebbistatin or cyclodextrin, a steep decrease in leukocyte adhesion was observed after an increase in the flow-rate from 0.25dyn/cm^2^ to 1.25dyn/cm^2^ (Fig. 9B). Since all inhibitors used in the flow assay reduced lateral mobility of clustered ICAM-1, we conclude that optimal ICAM-1 mobility is a prerequisite for ICAM-1 function to bind leukocytes to the endothelium under physiological flow conditions. The findings are summarized in figure 10.

## Discussion

Leukocytes bind through their integrins LFA-1/Mac-1 to endothelial ICAM-1 to establish firm adhesion. Upon leukocyte binding, ICAM-1 translocates to sites of adhesion, forming a typical ring structure around the adhered leukocyte [10], [19], [32], [33]. Here, we show that upon clustering ICAM-1 shifts to an immobile fraction in an actin-dependent manner, which also requires its intracellular domain. In addition, actin-regulating proteins such as Rac1 and myosin-II are involved in ICAM-1 dynamics, whereas the polymerization of actin and the formation of lipid rafts are necessary for the clustering.

Our recent work showed that filamin associates to ICAM-1 upon clustering [22]. In this report, we show that clustering of ICAM-1 not only recruits filamin, but also beta-actin, illustrated in figure 10. α-Actinin-1 and -4, other actin-binding proteins, are reported to bind to the intracellular domain of ICAM-1 [34]. In addition, these authors showed that α-actinin is required for proper leukocyte extravasation. Through this molecule, ICAM-1 may be linked to the actin cytoskeleton. The family of ERM-proteins has been implicated in the direct link of ICAM-1 to the actin cytoskeleton as well [17]. Deletion of ezrin results in reduced ICAM-1-positive filopodia. The authors furthermore show that deletion of the intracellular domain of ICAM-1 prevents the interaction with ezrin and also reduces filopodia. Our study indicates that deletion of the intracellular domain of ICAM-1 results in smaller and reduced numbers of filopodia. Adhesion of the leukocytes to tail-less ICAM-1 was not affected under static or low shear stress conditions. However, increasing the flow induces a loss of leukocyte adhesion to tail-less ICAM-1, but not full length ICAM-1 [17]. These data indicate that the intracellular domain plays an important role in ICAM-1 function, in line with previous reports [14], [15]. Our current data show that the intracellular domain of ICAM-1 is required for the shift to the immobile fraction. From these data, it is clear that the intracellular tail of ICAM-1, as well as the actin cytoskeleton is required for proper ICAM-1 clustering, in order to function efficiently as an adhesion molecule.

The small GTPase Rac1 and myosin-II activity are not only involved in ICAM-1 clustering, but appear to play a more general role in ICAM-1 dynamics. Interfering with the activity of one of these two molecules affects the lateral mobility of ICAM-1. Rac1 has not been implicated in ICAM-1 dynamics before, although many studies have shown involvement of Rac1 activity in membrane dynamics [28]. Interestingly, Heiska and colleagues showed that PIP2 is required for proper binding of the actin-binding protein ezrin to ICAM-1 [35]. Our lab and others have previously shown that Rac1 associates to PIP-5-Kinase, which may link Rac1 to PIP2-synthesis [36], [37]. This suggests that Rac1 is involved, through the regulation of inositol lipid turnover and the regulation of ezrin, in linking ICAM-1 to the actin cytoskeleton upon clustering. Myosin-II has not previously been implicated in ICAM-1 dynamics. However, the fact that ICAM-1 localizes at dorsal filopodia and moves within the lateral plane of the cells' plasma-membrane makes myosin-II a possible candidate to be involved in ICAM-1 dynamics. Recently, Khuon and colleagues showed that myosin-II in endothelial cells forms a ring-like structure that encapsulates an invading tumor cell [38]. Together these findings suggest that Rac1 and myosin-II activity are required for ICAM-1 dynamics.

The adhesive capacity of ICAM-1 can be tested using flow. By applying increasing flow rates on an anti-ICAM-1 antibody-coated bead bound to endothelium, the minimal flow rate that is required for proper adhesion by ICAM-1 can be determined. We have used beads instead of leukocytes for two reasons: First, the inhibitors used need to be present throughout the experiment. This may affect the leukocytes in their ability to bind to the endothelium. Second, leukocytes adhere not only to ICAM-1 but also to ICAM-2 and VCAM-1 and possibly other yet unidentified adhesion molecules on activated endothelial cells. Thus, to avoid confusion, we have used beads that specifically adhere to ICAM-1. Our results indicate that actin polymerization, Rac1 signaling and in particular myosin-II play a crucial role for optimal ICAM-1 adhesion (Fig. 10). The fact that actin polymerization is required for the shift of ICAM-1 to the immobile fraction may explain why ICAM-1 adhesion under flow is reduced following treatment of cells with cytochalasin B. Under these conditions, ICAM-1 is not efficiently anchored to the actin cytoskeleton which in turn results in lower adhesive capacity.

It has been reported that blocking myosin light chain kinase inhibits leukocyte transendothelial migration [39]. The authors suggested that MLC-kinase acts on the cell-cell junctions and by blocking its activity the junctions remain intact, resulting in a reduced passage of leukocytes. We show that myosin-II is recruited to sites of ICAM-1 adhesion and that blocking myosin-II activity results in an impaired ICAM-1-mediated adhesion under flow. In line with our data, Khuon and colleagues showed that myosin-II activity is found at sites of adhesion [38]. These data underscore the notion that ICAM-1 dynamics within the apical endothelial membrane are essential for optimal ICAM-1 adhesive function.

Yang and co-workers showed that Src and cortactin play an important role in the linking of ICAM-1 to the cytoskeleton [19]. These authors used pharmacological inhibitors to block Src activity. We choose to use MEF cells that lack three Src-like kinases Src, Yes and Fyn. Reconstitution of Src into these MEFs did not affect the ICAM-1 mobility in the plane of the membrane as determined by FRAP nor the adhesive function of ICAM-1. The latter finding is in line with the results reported by Yang and colleagues. Therefore, we concluded from these experiments that Src is not directly involved in the adhesive function of ICAM-1, but rather in transmitting intracellular signals downstream from ICAM-1 clustering, possibly to regulate cell-cell junction integrity [30].

In conclusion, we postulate that the dynamics of ICAM-1 within the plane of the membrane is vital for its function to induce firm adhesion under physiological flow conditions. Our data emphasize the contribution of ICAM-1 dynamics to its adhesive function and may therefore represent an interesting target for future therapies to reduce leukocyte accumulation to the vessel wall, as occurs in diseases such as atherosclerosis and rheumatoid arthritis.

## Supporting Information

Figure S1 ICAM-1-GFP co-localizes with endogenous ICAM-1 and associates to filamin and actin upon clustering. (A) TNF-α-stimulated endothelium was transiently transfected with ICAM-1-GFP, fixed and stained for ICAM-1-GFP in green, endogenous ICAM-1 in red. Merge shows co-localization in yellow and F-actin is in grayscale. Images were taken from the apical surface (upper panel) and at the baso-lateral plane (lower panel). Bars, 50 µm. (B) Beads, coated with ICAM-1 Ab, were allowed to adhere for 30 minutes to TNF-α-stimulated HUVECs. ICAM-1 was stained using an ICAM-1 Ab that is directly labelled with ALEXA 647. The results show that ICAM-1-GFP (green) and endogenous ICAM-1 (red) co-localize (Arrowheads; Merge in yellow) at sites of bead adhesion. DIC image shows localization of the beads. Images were taken from the apical surface (upper panel) and at the baso-lateral plane (lower panel). Bars, 10 µm. (C) ICAM-1-GFP was transfected into HeLa cells and anti-ICAM-1-coated magnetic beads were allowed to adhere for 30 minutes, resulting in clustering of ICAM-1, after which the cells were lysed ands treated as described in the legend of figure 2C. The images on the left show that the magnetic beads efficiently precipitated ICAM-1-GFP and that ICAM-1 clustering resulted in the recruitment of actin and filamin A. Images on the right show the expression of indicated proteins in the cell lysates. (D) ICAM-1-GFP was transfected into HeLa cells and anti-ICAM-1-coated magnetic beads were allowed to adhere for 5 or 20 minutes, as indicated. ICAM-1 was precipitated at both time points, whereas actin and filamin A and B were associated to ICAM-1 only after 20 minutes of clustering. Images on the right show the expression of indicated proteins in the cell lysates.
(0.29 MB TIF)

Figure S2 Rac1 activation downstream from ICAM-1 clustering. (A) ICAM-1 was clustered using anti-ICAM-1-antibody coated beads for 30 minutes on TNF-α-stimulated HUVECs. Rac1 activity was measured based on a CRIB-pull down assay from cell lysates. The results show that Rac1 is activated upon clustering of ICAM-1 (Upper panel). Lower panel shows equal levels of Rac1 in total cell lysates. (B) NSC-27632 inhibits Rac1 activity. HEK293 cells were transfected with GFP-Trio-D1, the first DH/PH domain of the guanine nucleotide exchange factor Trio and known to activate Rac1. The inhibitor (100 µM) is incubated for 30 minutes and showed that Trio-D1-induced Rac1 activity was reduced. Rac1 activity was measured using a CRIB-based pull down assay. The upper panel shows Rac1 activity, the middle panel depicts the total Rac1 protein levels in the total cell lysates and the lower panel shows the expression of the transfected Trio-D1 constructs, stained with an anti-GFP antibody.
(0.18 MB TIF)

Movie S1 Real-time recording of ICAM-1-GFP. HeLa cells were transfected with ICAM-1-GFP full length and real-time recording was taken from these cells for 45 minutes. Movie shows small filopodia at the apical surface of the cell.
(1.58 MB AVI)

## References

[1] Carman CV, Springer TA (2004). A transmigratory cup in leukocyte diapedesis both through individual vascular endothelial cells and between them.. J Cell Biol.

[2] Kluger MS (2004). Vascular endothelial cell adhesion and signaling during leukocyte recruitment.. Adv.Dermatol.

[3] van Buul JD, Kanters E, Hordijk PL (2007). Endothelial signaling by Ig-like cell adhesion molecules.. Arterioscler Thromb Vasc Biol.

[4] Vestweber D (2007). Molecular mechanisms that control leukocyte extravasation through endothelial cell contacts.. Ernst Schering Found Symp Proc.

[5] Wittchen ES (2009). Endothelial signaling in paracellular and transcellular leukocyte transmigration.. Front Biosci.

[6] Thompson PW, Randi AM, Ridley AJ (2002). Intercellular adhesion molecule (ICAM)-1, but not ICAM-2, activates RhoA and stimulates c-fos and rhoA transcription in endothelial cells.. J Immunol.

[7] van Buul JD, Voermans C, van den Berg V, Anthony EC, Mul FP (2002). Migration of human hematopoietic progenitor cells across bone marrow endothelium is regulated by vascular endothelial cadherin.. J Immunol.

[8] Burridge K, Wennerberg K (2004). Rho and Rac take center stage.. Cell.

[9] Etienne S, Adamson P, Greenwood J, Strosberg AD, Cazaubon S, Couraud PO (1998). ICAM-1 signaling pathways associated with Rho activation in microvascular brain endothelial cells.. J Immunol.

[10] van Buul JD, Allingham MJ, Samson T, Meller J, Boulter E (2007). RhoG regulates endothelial apical cup assembly downstream from ICAM1 engagement and is involved in leukocyte trans-endothelial migration.. J Cell Biol.

[11] Wojciak-Stothard B, Williams L, Ridley AJ (1999). Monocyte adhesion and spreading on human endothelial cells is dependent on Rho-regulated receptor clustering.. J Cell Biol.

[12] Tilghman RW, Hoover RL (2002). E-selectin and ICAM-1 are incorporated into detergent-insoluble membrane domains following clustering in endothelial cells.. FEBS Lett.

[13] Barreiro O, Yanez-Mo M, Serrador JM, Montoya MC, Vicente-Manzanares M (2002). Dynamic interaction of VCAM-1 and ICAM-1 with moesin and ezrin in a novel endothelial docking structure for adherent leukocytes.. J Cell Biol.

[14] Greenwood J, Amos CL, Walters CE, Couraud PO, Lyck R (2003). Intracellular domain of brain endothelial intercellular adhesion molecule-1 is essential for T lymphocyte-mediated signaling and migration.. J Immunol.

[15] Lyck R, Reiss Y, Gerwin N, Greenwood J, Adamson P, Engelhardt B (2003). T-cell interaction with ICAM-1/ICAM-2 double-deficient brain endothelium in vitro: the cytoplasmic tail of endothelial ICAM-1 is necessary for transendothelial migration of T cells.. Blood.

[16] Sans E, Delachanal E, Duperray A (2001). Analysis of the roles of ICAM-1 in neutrophil transmigration using a reconstituted mammalian cell expression model: implication of ICAM-1 cytoplasmic domain and Rho-dependent signaling pathway.. J Immunol.

[17] Oh HM, Lee S, Na BR, Wee H, Kim SH (2007). RKIKK motif in the intracellular domain is critical for spatial and dynamic organization of ICAM-1: functional implication for the leukocyte adhesion and transmigration.. Mol Biol Cell.

[18] Chen X, Kim TD, Carman CV, Mi LZ, Song G, Springer TA (2007). Structural plasticity in Ig superfamily domain 4 of ICAM-1 mediates cell surface dimerization.. Proc Natl Acad Sci USA.

[19] Yang L, Kowalski JR, Yacono P, Bajmoczi M, Shaw SK (2006). Endothelial cell cortactin coordinates intercellular adhesion molecule-1 clustering and actin cytoskeleton remodeling during polymorphonuclear leukocyte adhesion and transmigration.. J Immunol.

[20] Back AL, Gollahon KA, Hickstein DD (1992). Regulation of expression of the leukocyte integrin CD11a (LFA-1) molecule during differentiation of HL-60 cells along the monocyte/macrophage pathway.. J Immunol.

[21] Bohil AB, Robertson BW, Cheney RE (2006). Myosin-X is a molecular motor that functions in filopodia formation.. Proc Natl Acad Sci USA.

[22] Kanters E, van Rijssel J, Hensbergen PJ, Hondius D, Mul FP (2008). Filamin B mediates ICAM-1-driven leukocyte transendothelial migration.. J Biol Chem.

[23] van Buul JD, Hordijk PL (2009). Endothelial adapter proteins in leukocyte transmigration.. Thromb Haemost.

[24] Carman CV, Jun CD, Salas A, Springer TA (2003). Endothelial cells proactively form microvilli-like membrane projections upon intercellular adhesion molecule 1 engagement of leukocyte LFA-1.. J Immunol.

[25] Nakamura F, Osborn TM, Hartemink CA, Hartwig JH, Stossel TP (2007). Structural basis of filamin A functions.. J Cell Biol.

[26] Sverdlov M, Shinin V, Place AT, Castellon M, Minshall RD (2009). Filamin A Regulates Caveolae Internalization and Trafficking in Endothelial Cells.. Mol Biol Cell.

[27] Berg JS, Powell BC, Cheney RE (2001). A millennial myosin census.. Mol Biol Cell.

[28] Jaffe AB, Hall A (2005). Rho GTPases: biochemistry and biology.. Annu Rev Cell Dev Biol.

[29] Bouquier N, Vignal E, Charrasse S, Weill M, Schmidt S (2009). A cell active chemical GEF inhibitor selectively targets the Trio/RhoG/Rac1 signaling pathway.. Chem Biol.

[30] Allingham MJ, van Buul JD, Burridge K (2007). ICAM-1-mediated, Src- and Pyk2-dependent vascular endothelial cadherin tyrosine phosphorylation is required for leukocyte transendothelial migration.. J Immunol.

[31] Durieu-Trautmann O, Chaverot N, Cazaubon S, Strosberg AD, Couraud PO (1994). Intercellular adhesion molecule 1 activation induces tyrosine phosphorylation of the cytoskeleton-associated protein cortactin in brain microvessel endothelial cells.. J Biol Chem.

[32] Alcaide P, Auerbach S, Luscinskas FW (2009). Neutrophil recruitment under shear flow: it's all about endothelial cell rings and gaps.. Microcirculation.

[33] Millan J, Hewlett L, Glyn M, Toomre D, Clark P, Ridley AJ (2006). Lymphocyte transcellular migration occurs through recruitment of endothelial ICAM-1 to caveola- and F-actin-rich domains.. Nat Cell Biol.

[34] Celli L, Ryckewaert JJ, Delachanal E, Duperray A (2006). Evidence of a functional role for interaction between ICAM-1 and nonmuscle alpha-actinins in leukocyte diapedesis.. J Immunol.

[35] Heiska L, Alfthan K, Gronholm M, Vilja P, Vaheri A, Carpen O (1998). Association of ezrin with intercellular adhesion molecule-1 and -2 (ICAM-1 and ICAM-2). Regulation by phosphatidylinositol 4, 5-bisphosphate.. J Biol.Chem.

[36] Tolias KF, Rameh LE, Ishihara H, Shibasaki Y, Chen J (1998). Type I phosphatidylinositol-4-phosphate 5-kinases synthesize the novel lipids phosphatidylinositol 3,5-bisphosphate and phosphatidylinositol 5-phosphate.. J Biol Chem.

[37] van Hennik PB, ten Klooster JP, Halstead JR, Voermans C, Anthony EC (2003). The C-terminal domain of Rac1 contains two motifs that control targeting and signaling specificity.. J Biol Chem.

[38] Khuon S, Liang L, Dettman RW, Sporn PH, Wysolmerski RB, Chew TL (2010). Myosin light chain kinase mediates transcellular intravasation of breast cancer cells through the underlying endothelial cells: a three-dimensional FRET study.. J Cell Sci.

[39] Saito H, Minamiya Y, Saito S, Ogawa J (2002). Endothelial Rho and Rho kinase regulate neutrophil migration via endothelial myosin light chain phosphorylation.. J Leukoc Biol.

